# Acute recreational drug and ethanol poisoning among adolescents: An observational study from Oslo, Norway 2014–2023

**DOI:** 10.1016/j.toxrep.2025.102134

**Published:** 2025-09-30

**Authors:** Ingrid M. Stenbeck, Lüsette A. Subi, Mette Brekke, Odd Martin Vallersnes

**Affiliations:** a Faculty of Medicine, University of Oslo, PB 1078 Blindern, Oslo 0316, Norway; b Department of General Practice, University of Oslo, PB 1130 Blindern, Oslo 0318, Norway; c Oslo Accident and Emergency Outpatient Clinic, City of Oslo Health Agency, Trondheimsveien 233, Oslo 0477, Norway

**Keywords:** Adolescents, Alcohol, Cannabis, Ethanol, Recreational drugs, Benzodiazepines, Heroin

## Abstract

**Aims:**

To keep track on trends and changes over time, we describe drugs taken, clinical course, and incidence of recreational drug and ethanol poisoning among adolescents in Oslo, Norway, from 2014 to 2023.

**Methods:**

We included all patients ≤ 20 years presenting to the city’s main emergency primary care clinic with recreational drug poisoning in 2014–2023, from 2018 also including sole ethanol poisoning. Data were retrospectively collected from local patient records. Incidences were estimated per 1000 inhabitants in Oslo aged 10–20 years per year.

**Results:**

There were 1209 recreational drug poisonings, 51.1 % among girls. Cannabis was taken in 37.1 % of the cases, benzodiazepines in 22.2 %, heroin in 17.1 %, cocaine in 14.6 %, amphetamine in 13.1 %, and methylenedioxymethamphetamine (MDMA) in 12.2 %. More than one drug was taken in 406 (33.6 %) cases. There were 1675 sole ethanol poisonings, 59.9 % among girls. During the pandemic years 2020 and 2021 there was a temporary decrease in the incidence of sole ethanol poisoning, accompanied by an increase in recreational drug poisoning. Otherwise, the incidences were stable, except a marked increase for recreational drugs in 2023: recreational drug poisoning 1.20 (95 % CI 0.97–1.50) in 2014 and 3.55 (3.16–3.99) in 2023; sole ethanol poisoning 3.94 (3.50–4.42) in 2018 and 3.96 (3.54–4.42) in 2023. The incidence of recreational drug poisoning among girls were lower than among boys early in the study period, but higher from 2021.

**Conclusions:**

Recreational drug poisoning in Oslo among adolescents was dominated by ethanol, cannabis, benzodiazepines, and heroin.

## Highlights

• Adolescents are especially vulnerable to recreational drug and ethanol poisoning.
• Recreational drug poisoning in adolescents in Oslo, Norway, 2014–23, was recorded.
• The incidence of recreational drug poisoning among girls increased over the decade.
• Ethanol and cannabis were the most common substances taken.
• Benzodiazepines, cocaine, amphetamine, and MDMA were also frequent.

## Graphical Abstract

**Figure:**
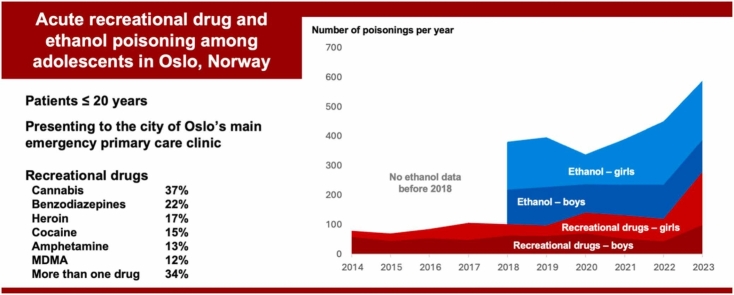

## 1 Introduction

Use of alcohol and recreational drugs are major causes of morbidity and early death [1]. In a 2019 survey among 15–16 year-olds in 35 European countries, 17 % reported ever using an illegal recreational drug, ranging from 4 % to 28 % across countries [2]. Cannabis was most commonly used, followed by amphetamine/methamphetamine, methylenedioxymethamphetamine (MDMA), and hallucinogens [2]. In Norway, 9 % reported ever using an illegal drug, most often cannabis [2]. As many as 79 % of 15–16 year-olds in the European study reported ever drinking alcohol, 33 % reported drinking for the first time at age 13 years or younger [2]. Among Norwegian 15–16 year-olds, 53 % reported ever drinking alcohol [2]. In a similar US study, 40 % reported ever using an illegal drug, again most commonly cannabis, and 43 % reported ever drinking alcohol [3].

Adolescence is a critical period of transition where recreational drug use may have serious impact on the evolving brain [4]. Adolescent drug use is associated with increased risk of mental health problems and addiction [4], [5]. Cannabis use is associated with increased risk of depression and suicide attempt [6]. Physical health problems are also more common among patients with a history of drug use during adolescence [5]. Learning and memory functions are affected by early exposure to recreational drugs [4], as is behavioural flexibility [7]. A possible explanation is that while a mature nervous system copes with moderate drug use through neuroadaptation, an immature nervous system incorporates these changes in its permanent pattern of reaction [4].

In a Norwegian study, 20-year mortality was five times higher than expected among patients treated for an acute poisoning [8]. However, in the younger age groups it was 10–16 times increased, making acute poisoning presentation to health services an important marker of hazardous use of recreational drugs and/or alcohol [8]. Detailed data on adolescent acute recreational drug toxicity presentations are sparse. Furthermore, trends in drug use continuously change. In a multi-centre study with data from 14 European countries, cannabis, new psychoactive substances, amphetamines, and cocaine were the drugs most often involved in presentations to emergency departments with acute recreational drug toxicity among adolescents [9]. Presentations involving alcohol only were not included in this study. In a previous study from Oslo, Norway, ethanol was by far the dominant agent among adolescents and young adults, followed by benzodiazepines, cocaine, MDMA, and hallucinogens [10].

### 1.1 Aims

We aim to describe acute poisoning with recreational drugs and/or ethanol among adolescents in Oslo, Norway, concerning

• age and sex,
• substances taken,
• clinical course, i.e., time and mode of presentation, length of stay, treatment given, and disposition,
• clinical features,
• time trends in incidence of poisoning.

## 2 Materials and methods

### 2.1 Design

The study was observational with retrospective registration of clinical data from electronic patient records.

### 2.2 Setting

The study was done at the Oslo Accident and Emergency Outpatient Clinic (OAEOC), the main emergency primary care clinic in Oslo (population 709,037 as per 1 January 2023), the capital city of Norway. The clinic serves the entire city at all hours and has about 200,000 consultations per year. Diagnostic and treatment resources are limited.

The Norwegian health system is two-tiered with a strong gate-keeping function. Patients must be assessed in primary care or by the ambulance service before transfer or referral to hospital. In Oslo, most patients with acute recreational drug or ethanol poisoning are managed in primary care, at the OAEOC [11].

### 2.3 Participants

We included all patients younger than 21 years presenting at the OAEOC from 1 January 2014–31 December 2023 with acute recreational drug poisoning. Using the definitions developed by the European Drug Emergencies Network (Euro-DEN), recreational drug poisoning encompassed poisoning with any psychoactive substance taken for recreational purposes [12]. Hence, poisoning with suicidal intention or inflicted by others were not included. Furthermore, in accordance with the Euro-DEN definitions, patients with sole ethanol poisoning were not included in the first part of the study period, though co-ingestion of ethanol was recorded. From 1 January 2018 we made a local expansion of the Euro-DEN inclusion criteria to also include sole ethanol poisoning.

Potentially eligible patients were identified from the contact reason noted in the registration lists in the electronic medical record system. The patient’s medical record was then reviewed to see whether the inclusion criteria were fulfilled.

### 2.4 Data collection and classification

Data were registered from the electronic patient records using the variable set developed by the Euro-DEN [12]. We registered age, sex, time of presentation, whether the patient was brought by ambulance, length of stay, disposition (medically discharged, transferred to hospital, admitted to psychiatric hospital, self-discharge, and death), toxic agents taken, clinical observations at presentation (respiratory rate, heart rate), clinical features during the episode (hyperthermia (temperature ≥ 39 °C), chest pain, palpitations, arrhythmia, hypertension (systolic blood pressure ≥ 180 mmHg), hypotension (systolic blood pressure ≤ 90 mmHg), vomiting, headache, seizures, agitation, anxiety, hallucinations, psychosis, lowest level of consciousness (measured by Glasgow Coma Scale (GCS)), and treatment (any treatment beyond mere observation, and treatment with sedation, naloxone, or flumazenil). For patients with sole ethanol poisoning, only a limited data set was collected (age, sex, time of presentation, length of stay, and disposition).

Data was collected by medical students trained for the task and supervised by the corresponding author.

The diagnosis of toxic agents taken was based on the assessment of the doctor treating the patient as registered in the medical record. As toxicological laboratory analyses are not done at the OAEOC, the doctor’s assessment was clinical and based on information from the patient and the patient’s companions and on the clinical presentation. Amphetamine and methamphetamine were co-categorized as amphetamine. Z-drugs were categorized as benzodiazepines.

We categorized age as < 16 years, 16–17 years, and 18–20 years. By Norwegian law, the age of majority is 18 years. However, for most health issues, the age of majority is 16 years. Furthermore, it is not legal to buy ethanol before turning 18 years.

When constructing the categorical variables tachypnoea (respiratory rate > 20/min), bradypnoea (respiratory rate < 10/min), tachycardia (heart rate ≥ 100/min), and bradycardia (heart rate < 50/min) from their respective continuous variables, missing values were treated as the clinical feature not being present. Data on lowest conscious level were not collected prior to October 2014, and when categorizing this variable (alert (GCS 15), drowsy (GCS 8–14), comatose (GCS 3–7)), missing values were treated as missing. Weekend presentation was defined as presenting between Saturday 00:00 and Sunday 23:59.

### 2.5 Statistics

Analyses were done using IBM SPSS version 29 or an online calculator from Epitools (http://epitools.ausvet.com.au). Comparisons of categorical variables were done using chi square test or Fisher’s exact test as appropriate. Comparisons of continuous variables were done using the non-parametric Kruskal-Wallis test. Incidence was estimated per 1000 inhabitants in Oslo aged 10–20 years (girls, boys, or total, as appropriate) per year [13]. 95 % confidence intervals (CI) for the insidences were estimated using the formula (e^ln incidence – 1.96^, e^ln incidence + 1,96^).

### 2.6 Ethics

The study was done as part of a quality improvement study, commissioned by the director of the Department of Emergency General Practice at the OAEOC and the Head of the Department of Acute Medicine at the Oslo University Hospital, as per the Norwegian Law on Health Personnel §26. The Oslo University Hospital Information Security and Privacy Office waived the need for approval by an ethics committee and the need for informed consent from the patients, in accordance with the Norwegian ethics committee regulations for quality improvement studies. Data were registered anonymously.

## 3 Results

### 3.1 Recreational drugs

From 2014–2023 there were 1209 recreational drug poisonings, 618 (51.1 %) among girls (Table 1). The patient was ≤ 15 years in 105 (8.7 %) cases, 16–17 years in 209 (17.3 %), and 18–20 years in 895 (74.0 %). The youngest patient was 12 years. There were more girls among the patients ≤ 15 years (81.0 % vs 54.1 % and 46.9 % in the older age groups, p < 0.001)). While treatment beyond mere observation was given to a larger proportion of patients 18–20 years (14.7 % vs 6.7 % and 7.2 % in the younger age groups, p = 0002), there was no difference across the age groups in the proportion transferred to hospital, in total 173 (14.3 %) patients.

**Table 1:** Recreational drug poisoning among adolescents in Oslo – clinical course.

	**≤ 15 years** **n (%)**	**16–17 years** **n (%)**	**18–20 years** **n (%)**	**Total** **n (%)**	**p-value**
**Girls**	85 (81.0)	113 (54.1)	420 (46.9)	618 (51.1)	**< 0.001**
**Weekend presentation**	37 (35.2)	69 (33.0)	391 (43.7)	497 (41.1)	**0.008**
**Brought by ambulance**	57 (54.3)	112 (53.6)	523 (58.4)	692 (57.2)	0.37
**Length of stay**^**a**^	2:46 (1:48–3:58)	2:29 (1:32–3:53)	2:58 (1:37–5:12)	2:50 (1:37–4:48)	0.017
**Treatment**^**b**^	7 (6.7)	15 (7.2)	132 (14.7)	154 (12.7)	**0.002**
**Sedation**	-	2 (1.0)	17 (1.9)	19 (1.6)	0.32
**Naloxone**	4 (3.8)	6 (2.9)	76 (8.5)	86 (7.1)	**0.007**
**Disposition**^**c**^					**0.002**
Medically discharged	84 (80.0)	146 (69.9)	590 (65.9)	820 (67.8)	**0.011**
Transferred hospital	16 (15.2)	35 (16.7)	122 (13.6)	173 (14.3)	0.49
Admitted psychiatric ward	3 (2.9)	5 (2.4)	40 (4.5)	48 (4.0)	0.32
Self-discharge	2 (1.9)	23 (11.0)	143 (16.0)	168 (13.9)	**< 0.001**
**Total**	105 (100)	209 (100)	895 (100)	1209 (100)	

Data from 2014 to 2023. Not including sole ethanol poisonings.

For comparisons and calculation of p-values, chi square test (or Fisher’s exact test when appropriate) were used for categorical variables and the non-parametric Kruskal-Wallis test for continuous variables.

^a^Median (interquartile range).

^b^Any treatment beyond mere observation.

^c^No patients died at the clinic.

Cannabis was the drug most commonly taken, reported in 448 (37.1 %) cases, followed by benzodiazepines in 269 (22.2 %), heroin in 207 (17.1 %), cocaine in 176 (14.6 %), amphetamine in 158 (13.1 %), and MDMA in 147 (12.2 %) (Table 2). More than one drug was taken in 406 (33.6 %) cases. Cannabis was reported in more than half the cases among patients ≤ 17 years. MDMA was more prevalent among patients ≤ 15 years (p < 0.001), while heroin, cocaine, and amphetamine were more prevalent among patients 18–20 years (p < 0.001, p = 0.014, and p < 0.001, respectively). Benzodiazepines were evenly distributed across the age groups. In 474 (39.2 %) cases the patient had ingested ethanol in addition to taking recreational drugs.

**Table 2:** Recreational drug poisoning among adolescents in Oslo – drugs taken.

	**≤ 15 years n (%)**	**16–17 years n (%)**	**18–20 years n (%)**	**Total n (%)**	**p-value**
**Cannabis**	56 (53.3)	108 (51.7)	284 (31.7)	448 (37.1)	**< 0.001**
**Benzodiazepines**	20 (19.0)	38 (18.2)	211 (23.6)	269 (22.2)	0.17
**Heroin**	4 (3.8)	20 (9.6)	183 (20.4)	207 (17.1)	**< 0.001**
**Cocaine**	10 (9.5)	20 (9.6)	146 (16.3)	176 (14.6)	**0.014**
**Amphetamine**	9 (8.6)	12 (5.7)	137 (15.3)	158 (13.1)	**< 0.001**
**MDMA**	25 (23.8)	26 (12.4)	96 (10.7)	147 (12.2)	**< 0.001**
**Other opioids**	7 (6.7)	6 (2.9)	62 (6.9)	75 (6.2)	0.089
**GHB**	3 (2.9)	12 (5.7)	57 (6.4)	72 (6.0)	0.35
**Hallucinogens**	5 (4.8)	15 (7.2)	48 (5.4)	68 (5.6)	0.55
**Other stimulants**	4 (3.8)	10 (4.8)	23 (2.6)	37 (3.1)	0.22
**Other/Unknown**	13 (12.4)	17 (8.1)	111 (12.4)	141 (11.7)	0.22
**More than one drug**^**a**^	33 (31.4)	51 (24.4)	322 (36.0)	406 (33.6)	**0.005**
**Ethanol + any drug(s)**	33 (31.4)	68 (32.5)	373 (41.7)	474 (39.2)	**0.012**
**Total**	105 (100)	209 (100)	895 (100)	1209 (100)	

Data from 2014 to 2023. Not including sole ethanol poisonings.

For comparisons and calculation of p-values, chi square test (or Fisher’s exact test when appropriate) was used.

^a^Not including ethanol. Among total number of recreational drug poisonings: two drugs: 247 (20.4 %), three drugs: 107 (8.9 %), four or more drugs: 52 (4.3 %).

Percentages for individual drugs add up to more than 100 % as more than one drug was taken in 33.6 % of the cases.

GHB: gamma-hydroxybutyrate; MDMA: methylenedioxymethamphetamine.

Apart from a reduced conscious level being more prevalent among the older patients (p = 0.013) and vomiting more prevalent among the younger (p < 0.001), there were only minimal differences across the age groups concerning clinical features (Table 3).

**Table 3:** Recreational drug poisoning among adolescents in Oslo – clinical features.

	**≤ 15 years n (%)**	**16–17 years n (%)**	**18–20 years n (%)**	**Total n (%)**	**p-value**
**Tachypnoea***(RR > 20/min)*^*a*^	4 (3.8)	20 (9.6)	57 (6.4)	81 (6.7)	0.12
**Bradypnoea***(RR < 10/min)*^*a*^	-	1 (0.5)	16 (1.8)	17 (1.4)	0.22
**Tachycardia***(HR ≥ 100/min)*^*a*^	52 (49.5)	92 (44.0)	379 (42.3)	523 (43.3)	0.36
**Bradycardia***(HR < 50/min)*^*a*^	1 (1.0)	4 (1.9)	1 (0.1)	6 (0.5)	**0.004**
**Hypertension***(SBP ≥ 180 mmHg)*	-	1 (0.5)	-	1 (0.1)	0.26
**Hypotension***(SBP ≤ 90 mmHg)*	1 (1.0)	5 (2.4)	15 (1.7)	21 (1.7)	0.69
**Chest pain**	4 (3.8)	17 (8.1)	72 (8.0)	93 (7.7)	0.30
**Palpitations**	11 (10.5)	23 (11.0)	66 (7.4)	100 (8.3)	0.16
**Arrhythmias**	-	-	2 (0.2)	2 (0.2)	1.00
**Hyperthermia***(temp ≥ 39 °C)*	-	-	3 (0.3)	3 (0.2)	1.00
**Headache**	7 (6.7)	8 (3.8)	30 (3.4)	45 (3.7)	0.24
**Vomiting**	29 (27.6)	43 (20.6)	123 (13.7)	195 (16.1)	**< 0.001**
**Seizures**	1 (1.0)	2 (1.0)	14 (1.6)	17 (1.4)	0.91
**Agitation**	14 (13.3)	29 (13.9)	139 (15.5)	182 (15.1)	0.73
**Anxiety**	9 (8.6)	34 (16.3)	139 (15.5)	182 (15.1)	0.15
**Hallucinations**	6 (5.7)	16 (7.7)	67 (7.5)	89 (7.4)	0.79
**Psychosis**	2 (1.9)	10 (4.8)	52 (5.8)	64 (5.3)	0.22
**Lowest conscious level**^**b**^					**0.044**
*Alert (GCS 15)*	61 (59.2)	107 (54.6)	396 (46.6)	564 (49.1)	**0.013**
*Drowsy (GCS 8–14)*	37 (35.9)	79 (40.3)	385 (45.3)	501 (43.6)	0.12
*Comatose (GCS 3–7)*	5 (4.9)	10 (5.1)	69 (8.1)	84 (7.3)	0.21
**Total**	105 (100)	209 (100)	895 (100)	1209 (100)	

Data from 2014 to 2023. Not including sole ethanol poisonings.

For comparisons and calculation of p-values, chi square test (or Fisher’s exact test when appropriate) was used.

^a^At presentation.

^b^Missing value in 60 cases, total n = 1149.

GCS: Glasgow Coma Scale score; HR: heart rate; RR: respiratory rate; SBP: systolic blood pressure; temp: temperature.

### 3.2 Ethanol

From 2018–2023 there were 1675 poisonings with ethanol only, 1004 (59.9 %) among girls (Table 4). The patient was ≤ 15 years in 147 (8.8 %) cases, 16–17 years in 292 (17.4 %), and 18–20 years in 1236 (73.8 %). The youngest patient was 11 years. Sex was evenly distributed across the age groups. In 27 (1.6 %) cases the patient was transferred to hospital, more often among the patients ≤ 15 years (6.1 % vs 1.7 % and 1.1 % in the older age groups, p < 0.001).

**Table 4:** Poisoning with ethanol only among adolescents in Oslo – clinical course.

	**≤ 15 years** **n (%)**	**16–17 years** **n (%)**	**18–20 years** **n (%)**	**Total** **n (%)**	**p-value**
**Girls**	98 (66.7)	165 (56.5)	741 (60.0)	1004 (59.9)	0.12
**Weekend presentation**	68 (46.3)	153 (52.4)	796 (64.4)	1017 (60.7)	**< 0.001**
**Length of stay**^**a**^	2:53 (1:52–4:21)	3:01 (1:58–4:41)	3:42 (1:57–5:09)	3:25 (1:57–5:02)	**0.002**
**Disposition**^**b**^					**< 0.001**
Medically discharged	133 (90.5)	271 (92.8)	1100 (89.0)	1504 (89.8)	0.15
Transferred hospital	9 (6.1)	5 (1.7)	13 (1.1)	27 (1.6)	**< 0.001**
Admitted psychiatric ward	1 (0.7)	1 (0.3)	3 (0.2)	5 (0.3)	0.39
Self-discharge	4 (2.7)	15 (5.1)	120 (9.7)	139 (8.3)	**0.001**
**Total**	147 (100)	292 (100)	1236 (100)	1675 (100)	

Data from 2018 to 2023. Sole ethanol poisonings only.

For comparisons and calculation of p-values, chi square test (or Fisher’s exact test when appropriate) was used for categorical variables and the non-parametric Kruskal-Wallis test for continuous variables.

^a^Median (interquartile range).

^b^No patients died at the clinic.

### 3.3 Time trends

The incidence of recreational drug poisoning was 1.20 per 1000 inhabitants (95 % CI 0.97–1.50) in 2014 and remained stable, except a temporary increase during the covid pandemic years of 2020 and 2021, peaking at 1.90 (1.61–2.24) in 2020, until a marked increase in 2023 to 3.55 (3.16–3.99) (Table 5). The incidence of sole ethanol poisoning was 3.94 (3.50–4.42) in 2018 and remained stable except a temporary decrease during the covid pandemic years, with a low point at 2.66 (2.31–3.06) in 2020. The incidence of recreational drug poisoning was lower among girls than boys in the early part of the ten-year-period, equal in 2017 and 2020, then higher from 2021 to 2023. The incidence of sole ethanol poisoning was consistently higher among girls than boys, except in 2020.

**Table 5:** Time trends in incidence (95 % confidence interval) of recreational drug poisoning among adolescents in Oslo – drugs taken.

		**2014**	**2015**	**2016**	**2017**	**2018**	**2019**	**2020**	**2021**	**2022**	**2023**
**Girls**	*Recreational drugs*^*a*^	0.65 (0.42–1.00)	0.75 (0.51–1.11)	0.91 (0.64–1.29)	1.70 (1.31–2.19)	1.07 (0.78–1.47)	1.00 (0.72–1.39)	1.93 (1.53–2.44)	2.13 (1.71–2.65)	2.02 (1.62–2.52)	4.62 (4.00–5.35)
	*Ethanol only*^*b*^	-	-	-	-	4.60 (3.95–5.36)	4.71 (4.05–5.47)	2.75 (2.26–3.34)	4.12 (3.52–4.82)	5.64 (4.93–6.44)	5.16 (4.50–5.93)
	*Total*	-	-	-	-	5.67 (4.94–6.51)	5.71 (4.98–6.55)	4.68 (4.03–5.43)	6.25 (5.50–7.10)	7.66 (6.83–8.58)	9.79 (8.86–10.82)
**Boys**	*Recreational drugs*^*a*^	1.74 (1.35–2.25)	1.33 (0.99–1.78)	1.56 (1.20–2.04)	1.34 (1.01–1.78)	1.75 (1.37–2.24)	1.67 (1.30–2.14)	1.88 (1.48–2.37)	1.38 (1.05–1.81)	1.12 (0.83–1.51)	2.49 (2.04–3.03)
	*Ethanol only*^*b*^	-	-	-	-	3.29 (2.74–3.93)	3.56 (3.00–4.22)	2.57 (2.11–3.14)	2.73 (2.25–3.31)	3.00 (2.50–3.60)	2.76 (2.29–3.33)
	*Total*	-	-	-	-	5.04 (4.36–5.83)	5.23 (4.54–6.02)	4.45 (3.82–5.18)	4.11 (3.51–4.81)	4.12 (3.53–4.81)	5.25 (4.58–6.01)
**Total**^**c**^	*Recreational drugs*^*a*^	1.20 (0.97–1.50)	1.04 (0.83–1.32)	1.24 (1.00–1.53)	1.52 (1.25–1.83)	1.42 (1.16–1.72)	1.34 (1.10–1.63)	1.90 (1.61–2.24)	1.75 (1.48–2.08)	1.57 (1.31–1.88)	3.55 (3.16–3.99)
	*Ethanol only*^*b*^	-	-	-	-	3.94 (3.50–4.42)	4.13 (3.69–4.62)	2.66 (2.31–3.06)	3.42 (3.03–3.87)	4.31 (3.87–4.81)	3.96 (3.54–4.42)
	*Total*	-	-	-	-	5.35 (4.84–5.92)	5.47 (4.96–6.03)	4.56 (4.10–5.07)	5.18 (4.69–5.71)	5.88 (5.37–6.45)	7.50 (6.92–8.13)
	***Specific drugs***										
	*Cannabis*	0.43 (0.29–0.62)	0.43 (0.30–0.62)	0.42 (0.29–0.61)	0.60 (0.44–0.81)	0.63 (0.47–0.84)	0.55 (0.41–0.75)	0.69 (0.52–0.91)	0.56 (0.41–0.75)	0.61 (0.46–0.82)	1.21 (0.99–1.48)
	*Benzodiazepines*	0.26 (0.16–0.42)	0.10 (0.05–0.22)	0.29 (0.19–0.45)	0.29 (0.18–0.44)	0.28 (0.18–0.43)	0.19 (0.11–0.33)	0.31 (0.21–0.47)	0.40 (0.28–0.57)	0.37 (0.25–0.53)	1.15 (0.93–1.41)
	*Heroin*	0.23 (0.14–0.38)	0.10 (0.05–0.22)	0.31 (0.20–0.47)	0.30 (0.20–0.46)	0.38 (0.26–0.55)	0.11 (0.06–0.22)	0.20 (0.21–0.34)	0.32 (0.21–0.48)	0.22 (0.14–0.36)	0.66 (0.51–0.87)
	*Amphetamine*	0.23 (0.14–0.38)	0.21 (0.12–0.35)	0.23 (0.14–0.38)	0.23 (0.14–0.37)	0.15 (0.09–0.28)	0.15 (0.08–0.27)	0.34 (0.23–0.50)	0.28 (0.18–0.43)	0.14 (0.08–0.26)	0.23 (0.14–0.36)
	*Cocaine*	0.06 (0.02–0.16)	0.09 (0.04–0.20)	0.13 (0.07–0.25)	0.09 (0.04–0.19)	0.17 (0.10–0.30)	0.21 (0.12–0.34)	0.14 (0.07–0.25)	0.36 (0.25–0.52)	0.43 (0.31–0.61)	0.69 (0.53–0.90)
	*MDMA*	0.03 (0.01–0.12)	0.07 (0.03–0.18)	0.10 (0.05–0.21)	0.20 (0.12–0.34)	0.11 (0.06–0.22)	0.18 (0.10–0.31)	0.34 (0.23–0.50)	0.23 (0.14–0.36)	0.17 (0.10–0.29)	0.55 (0.41–0.74)

Data from 2018 to 2023. All cases included.

Incidences were estimated per 1000 inhabitants (girls, boys, or total, as appropriate) in Oslo aged 10–20 years per year.

^a^With or without ethanol.

^b^Data not available for poisoning with ethanol only for 2014–2017.

^c^Total, girls and boys.

MDMA: methylenedioxymethamphetamine.

Across the decade from 2014 to 2023, the incidence of poisoning with cannabis, benzodiazepines, and heroin were fairly stable, until markedly increasing in 2023 (Table 5). The same trend was seen for MDMA poisoning, with an additional peak in 2020. The incidence of cocaine poisoning gradually increased already from 2021, while the incidence of amphetamine poisoning remained stable across the decade.

## 4 Discussion

Ethanol was by far the most common substance taken. The younger patients were more often girls and more often had taken cannabis and MDMA, while heroin, cocaine, and amphetamine were more prevalent among patients 18 years and older. The incidence of recreational drug and ethanol poisoning was generally stable through the study period, until a marked increase for recreational drugs in 2023. During the pandemic years 2020 and 2021 there was a temporary decrease in the incidence of ethanol poisoning, accompanied by an increase in recreational drug poisoning, most markedly MDMA. The incidence of recreational drug poisoning among girls steadily increased through the study period.

### 4.1 Age and sex

There were few patients below the age of 18 years. Most likely because 18 years is the age of majority in Norway. It is also the legal age limit for buying alcohol. Combining ethanol and recreational drugs was also more common after turning 18 years, not surprising as ethanol then becomes legal and hence more available, and in line with findings from European emergency departments [9]. As minors would not be allowed to enter bars and clubs, they would mostly drink ethanol in private homes. Personnel in the nightlife economy would probably have a lower threshold for calling an ambulance when encountering an ethanol poisoning than minors partying at home.

Half the recreational drug poisonings in our adolescent population were among girls, in contrast to the 23–32 % female proportion commonly reported in adult patients in emergency departments both in Norway and other European countries [9], [11], [14], but more in line with studies on adolescents from Canada, Australia, and the USA reporting 31–56 % girls [15], [16], [17], [18], [19]. However, in a recent report from Oslo high schools, 40–50 % more boys than girls had used cannabis the last year, and twice as many boys had used other illegal drugs [20]. This is in contrast to our finding of a higher and increasing incidence of recreational drug poisoning among girls from 2021, for which we have no clear explanation. In line with our findings, sole ethanol poisoning has been found to be more evenly distributed between girls and boys [21], [22], [23], [24]. The European Monitoring Centre for Drugs and Drug Addiction (EMCDDA) reports that fewer Norwegian adolescent girls use recreational drugs but more drink alcohol compared to other European countries [2].

### 4.2 Recreational drugs

Ethanol aside, cannabis was the most prevalent drug taken, especially among patients younger than 18 years. Heroin, cocaine, and amphetamine were more prevalent among patients 18 years and older. This is again in line with findings from European emergency departments and may indicate a change to heavier drugs with increasing age [9]. However, hallucinogens and new psychoactive substances were more prevalent among the younger patients elsewhere in Europe [9]. It is highly likely that new psychoactive substances were underreported in our study, as registration of toxic agents was based on the clinical assessment of the doctor treating the patient. Some new psychoactive substances may have been diagnosed as unspecified stimulants or hallucinogens, or as their more classical relatives, e.g. amphetamine or MDMA. Furthermore, what was taken by the patient may have contained other substances than they were told. In a previous study in Oslo, we found new psychoactive substances in the blood sample of 13 % of the cases, though not reported by the patient nor suspected by the doctor [25]. A Canadian study found more MDMA with increasing age [15], in contrast to our finding that MDMA was more prevalent among the youngest patients. In our study 33.6 % of the patients taking recreational drugs had taken more than one drug, quite similar to the 37.9 % reported in adult populations presenting to European emergency departments [9].

The high prevalence of cannabis poisoning is not surprising as it is the most commonly used illegal drug in Norway, with 6 % of girls and 11 % of boys 15–16 years of age reporting ever using cannabis, increasing to 20 % in the age group 15–20 years [2]. Among 15–20 year olds in Oslo, as many as 30 % report ever using cannabis [26]. In comparison, only 0.9–1.7 % report ever using amphetamine, ecstasy, cocaine, gamma-hydroxybutyrate (GHB), heroin, or hallucinogens [2]. As many as 4.5 % report ever using an inhalant and 3.1 % a new psychoactive substance [2], again interestingly not showing up in our study, probably due to underdiagnosing, cf. the previous paragraph [25].

### 4.3 Ethanol

Nearly 80 % of the cases involved ethanol and two out of three cases were due to ethanol only. This is similar to findings from emergency departments in Australia and Canada and from US ambulance services, though fewer patients combined ethanol with other drugs in our study [15], [16], [18].

The proportion of Norwegian 15–16 year olds having ever used alcohol has been decreasing since the turn of the century [2], [27]. Excepting the pandemic years, the number of adolescent patients treated for ethanol poisoning increased through our study period (though the incidence was stable) and was higher than in studies from 2004 and 2012 [21], [28]. A possible explanation is that though fewer adolescents drink, the ones who do drink more [29].

### 4.4 Clinical course and clinical features

There were hardly any differences in the severity of poisoning across the age groups, except a larger proportion of older patients presenting with reduced conscious level. The clinical features were as expected from the involved drugs; unspecific tachycardia and vomiting; reduced conscious level associated with cerebral depressants such as ethanol, benzodiazepines, and heroin; agitation, anxiety, palpitations, and chest pain associated with stimulants such as amphetamine, cocaine, and MDMA; hallucinations and psychosis associated with hallucinogens, cannabis, and amphetamine. We have not found studies on hospital admission for recreational drug poisoning in comparable age groups, but the 14 % transferred to somatic hospital departments among our adolescent patients is slightly lower than the 15–20 % reported among adults, and the 4 % transferred to a psychiatric ward is similar to the 2–5 % among adults [11], [30]. Only 2 % of the patients with ethanol only poisoning were transferred to hospital, considerably fewer than the 10 % of adolescents with ethanol poisoning admitted from the emergency department in a Spanish hospital study [22]. However, our emergency primary care population do not encompass the more severe cases brought directly to hospital emergency departments by the ambulance service in Oslo.

### 4.5 Time trends

Though mostly stable from 2014 to 2022, there was a marked increase in the incidence of recreational drug poisoning among adolescents in 2023, possibly representing a continuation of the increase from 2006 to 2015 previously found in a nationwide registry study [31]. During the pandemic years there was a marked reduction in the incidence of sole ethanol poisoning, probably due to the shut-down of restaurants and the club scene. Simultaneously, there was a marked increase in the incidence of recreational drug poisoning. We have no obvious explanation for this, nor for the increase in the number of recreational drug poisonings in 2023. The increasing incidence of cocaine poisoning from 2021 is also seen elsewhere in Europe and probably reflects increased availability and more widespread use [1], [32]. There were no changes in the severity of the poisonings across the decade, judged by clinical features and hospital transfer rates.

### 4.6 Strengths and limitations

Our data material is large and covers ten years. Though the more severe cases of poisoning are brought directly to hospital by the ambulance service, and a significant number of patients with heroin poisoning decline transport to hospital or the OAEOC after ambulance treatment, the large majority of patients with recreational drug or ethanol poisoning in Oslo are treated at the OAOEC [11]. Hence, our results should be informative of the situation and trends in a Northern European capital city concerning which recreational drugs are involved in poisoning needing emergency health care attention.

We did not register the identity of the included patients. Hence, we do not know how many patients had several presentations during the study period, who would constitute a group with increased risk of morbidity, substance use disorders, and premature death [8], [29].

As the cases were included retrospectively based on the reason for contact noted in the patient registration lists, some may have been missed. However, we do not have reason to believe that this would be many. Furthermore, there may be some inter-rater variability in inclusion and data registration.

There is some uncertainty concerning the diagnosis of the drugs involved in the poisonings as toxicological testing was not done. However, the clinical assessment was based on all information available to the doctor treating the patient, combining assessment of the clinical presentation with any reports on toxic agents taken. Studies comparing clinical assessment with toxicological testing find that patients generally have taken the drugs suspected, though clinical assessment often underestimates the number of drugs taken [33]. Hence, we find it likely that some of our patients had taken more drugs than reported. They may also have reported more commonly used drugs instead of new psychoactive substances, as what they were taking may have contained other drugs than they thought [25]. However, clinical assessment of drugs taken is the mainstay of acute management of poisoned patients, and the diagnoses we registered were used to guide real clinical decisions.

## 5 Conclusion

Not surprisingly, ethanol was by far the most common psychoactive substance involved in acute poisoning among adolescents, while cannabis was the most commonly involved recreational drug. More than half the patients were girls, and the incidence of recreational drug poisoning among girls increased through the decade. The increasing incidence of cocaine poisoning is worrying, as are the general increase in recreational drug poisoning in 2023 and the high number of heroin poisonings among 18–20 year olds. Though the reported use of ethanol and recreational drugs among Norwegian adolescents is decreasing, the sustained and lately increasing incidence of poisoning may reflect a subgroup with hazardous ethanol and substance use.

## Author statements

na

## Funding sources

This research did not receive any specific grant from funding agencies in the public, commercial, or not-for-profit sectors.

## CRediT authorship contribution statement

**Lüsette A Subi:** Writing – original draft, Visualization, Investigation, Formal analysis, Conceptualization. **Mette Brekke:** Writing – review & editing, Supervision, Resources, Conceptualization. **Ingrid M Stenbeck:** Writing – original draft, Visualization, Investigation, Formal analysis, Conceptualization. **Odd Martin Vallersnes:** Writing – review & editing, Writing – original draft, Visualization, Validation, Supervision, Resources, Project administration, Methodology, Investigation, Formal analysis, Data curation, Conceptualization.

## Declaration of Competing Interest

The authors declare that they have no known competing financial interests or personal relationships that could have appeared to influence the work reported in this paper

## Data Availability

The dataset for the current study is not publicly available as several manuscripts based on the larger Euro-DEN dataset are in preparation. The dataset is available on reasonable request.
